# Bringing paediatric coronary artery abnormalities into view: the role of virtual reality

**DOI:** 10.1093/ehjimp/qyag151

**Published:** 2026-08-31

**Authors:** Carol Wing-Kei Ng, Maggie Yin-Ting Chiu, Samuel Chung-Sum Ho, Elaine Yee-Ling Kan, Yiu-Fai Cheung

**Affiliations:** Department of Radiology, Hong Kong Children’s Hospital, Hong Kong, China; Department of Radiology, Hong Kong Children’s Hospital, Hong Kong, China; Department of Paediatrics and Adolescent Medicine, Hong Kong Children’s Hospital, Hong Kong, China; Department of Radiology, Hong Kong Children’s Hospital, Hong Kong, China; Department of Paediatrics and Adolescent Medicine, Hong Kong Children’s Hospital, Hong Kong, China; Department of Paediatrics and Adolescent Medicine, School of Clinical Medicine, Li Ka Shing Faculty of Medicine, The University of Hong Kong, Hong Kong, China

**Keywords:** virtual reality, congenital imaging, coronary, computed tomography

## Abstract

**Purpose:**

Virtual reality (VR) is increasingly being used to present imaging data of congenital cardiac cases for surgical and interventional planning, as well as for education. This study aims to explore the use of VR in illustrating coronary artery anomalies, leveraging its capability for interactive three-dimensional visualization and manipulation.

**Methodology:**

Seven paediatric cases with coronary artery pathologies were selected, representing both congenital and acquired anomalies. CT coronary angiography datasets were processed using the commercially available VR software Elucis for segmentation and visualization of coronary anatomy.

**Results:**

The study cohort comprised of seven paediatric patients, four infants (aged 2 days–11 months) with cardiac diagnoses of TGA with single coronary origin, ALCAPA (Anomalous Left Coronary Artery from the Pulmonary Artery), Kawasaki disease with right coronary artery aneurysm, Sinus of Valsalva aneurysm; and three teenagers (aged 13–16 years) with ALCAPA, Kawasaki disease with left anterior descending(LAD) artery aneurysm and Kawasaki disease with obstructed LAD and a LIMA(left internal mammary artery) graft. We were able to depict the coronary artery origins in all 7 cases with VR. Coronary artery aneurysms were well demonstrated in all the Kawasaki cases. Large collaterals in the ALCAPA were well shown by VR. The spatial relationship between the Sinus of Valsalva aneurysm and the coronary arteries were well demonstrated. Teenagers had larger coronaries and segmentation time used was less than that of infants (2 h vs. 1 h).

**Conclusion:**

VR of paediatric coronary anomalies is feasible and provides a practical tool for pre-surgical planning, interventional strategy development, and medical education.

## Graphical Abstract

**Graphical Abstract:**
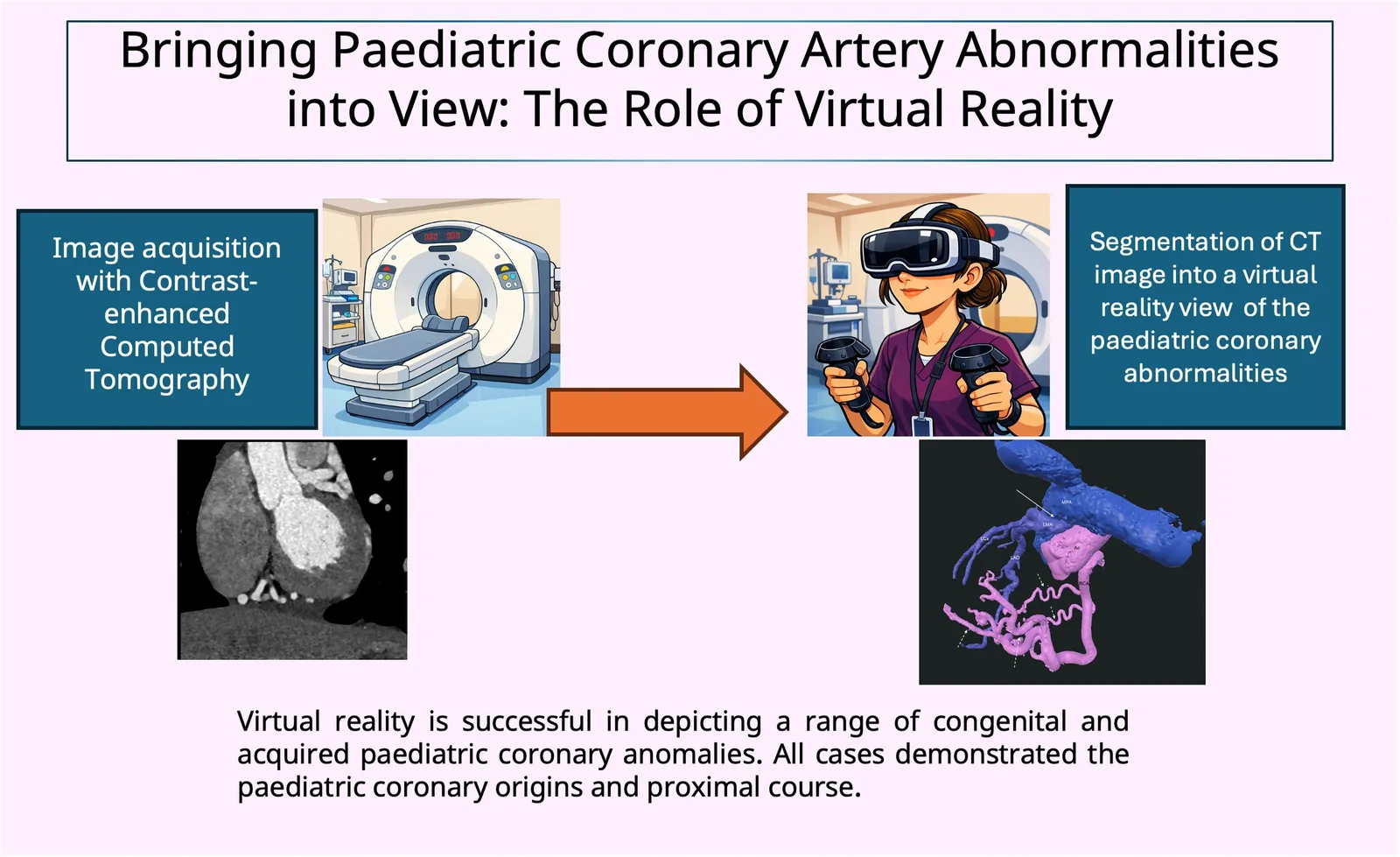

## Introduction

Virtual reality (VR) is increasingly used in congenital cardiac imaging for interactive patient- specific 3D visualisation for multidisciplinary case review, education, and procedural planning.^1–3^ The VR platforms reduce the cognitive burden of inferring complex three-dimensional relationships from multiple two-dimensional image planes and allow more intuitive exploration of the anatomy.^1^

Most published VR applications in congenital heart disease have, however, focused on intracardiac anatomy and larger vessels.^1–3^ Coronary-specific experience remains limited and largely case-based, including VR-assisted coronary bypass planning in Kawasaki disease and visualisation of anomalous right coronary artery arising from the pulmonary trunk.^4,5^ The feasibility and clinical role of VR across a broader spectrum of paediatric coronary abnormalities remain insufficiently described.

Application of VR to paediatric coronary arteries presents distinct technical challenges. Accurate depiction of vessels measuring only a few millimetres depends on adequate source data CT scanning technique with high spatial and temporal resolution, adequate contrast opacification and minimal cardiac motion. Segmentation of such small structures is also time-intensive and require substantial manual refinement. These technical demands may partly explain the relative scarcity of paediatric coronary applications.

In our institutional workflow, the primary intended role of VR is for multidisciplinary anatomical review of coronary origin, aneurysm morphology, collateral pathways and relationships to adjacent cardiovascular structures and facilitation of communication and discussion among radiologists, cardiologists and surgeons.^2,3^ Preprocedural planning is of particular relevance in selected cases in which these spatial relationships may influence surgical or interventional strategy, while education is considered an important secondary benefit. We therefore evaluated the technical feasibility of CT-derived VR rendering across a spectrum of congenital and acquired paediatric coronary abnormalities.

## Methods

### Study design

This pilot study included 7 children, 4 infants and 3 teenagers, with congenital and acquired coronary abnormalities, selected from our institutional archive of CT performed between October 2020 and December 2025. The inclusion of both congenital and acquired lesions enabled the assessment of VR applications in the identification of coronary ostium, assessment of the early vessel course, and depiction of aneurysm and collateral formation. Ethical approval with waiving of the need for patient consent was obtained. Contrast-enhanced source CT datasets were acquired from a dual-source scanner (SOMATOM Force Siemens Healthineers, Erlangen, Germany) with a 0.25 s gantry rotation and 66 ms temporal resolution. Acquisition was tailored to age and heart rate. Retrospective ECG gating in 4 infants permitted multiphase reconstruction and selection of the phase with least coronary motion, whereas prospective ECG triggered sequential acquisition (*n* = 2) or high pitch dual source helical acquisition (*n* = 1) was used in teenagers. General anaesthesia with breathing suspension minimized breathing motion in infants while teenagers were asked to hold their breaths and scanned awake.

### VR processing and assessment

The most motion-free cardiac phase with optimal coronary definition was selected by a radiologist specializing in congenital cardiac imaging on a PACS software Syngo.Via, Version VB60 (Siemens Healthcare GmBH, Erlangen, Germany). The CT DICOM datasets were then imported into Elucis VR (Realize Medical, Ottawa, Canada). Segmentation was performed by an experienced radiographer and verified by the radiologist. Segmentation was focused on the coronary arteries and adjacent cardiovascular structures to provide the anatomical context. A threshold-based tool was first used to select high-attenuation cardiac chambers and great vessels. Manual editing was then used to separate the chambers, great vessels, and coronary arteries. For small-sized coronary arteries in neonates and distal coronary artery segments in teenagers not adequately captured by thresholding alone, manual refinement was performed using the add, subtract, grow, and smoothing functions. Surrounding structures including aortic root, pulmonary artery, and cardiac chambers were included to enable the interpretation of coronary findings in the true spatial setting rather than as isolated vessel renderings. The generated models were reviewed in VR using a Meta Quest 3 headset, which enabled rotation, magnification, and inspection from multiple angles. Relevant cardiovascular structures were segmented and colour-coded to aid spatial orientation and inform lesion-specific anatomy. The primary assessment outcome was technical feasibility, defined as successful creation of a VR rendering displaying the coronary anatomy of interest. Secondary assessment outcomes included clear description of specific lesions (coronary origin and proximal course, aneurysm morphology, collateral depiction) and time taken for segmentation.

## Results

**Figure 1:**
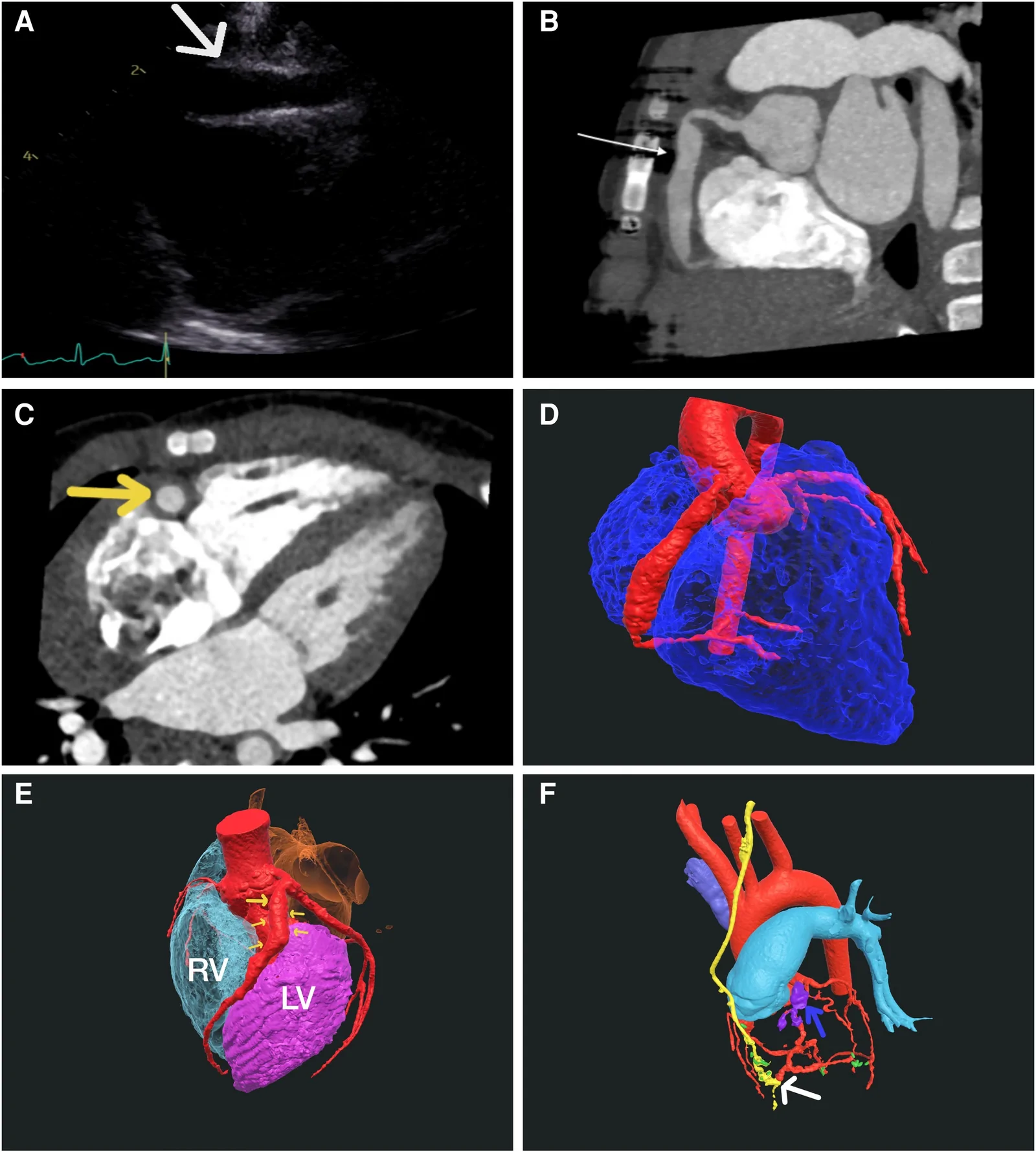
Mapping the coronary aneurysm landscape in Kawasaki disease. An 8-month-old boy with Kawasaki disease shown in panels *A–D*. (*A*) Transthoracic echocardiography shows a giant right coronary artery (RCA) aneurysm (solid white arrow). (*B*, *C*) Computed tomography (CT) angiogram demonstrates the full longitudinal extent of the giant RCA aneurysm (white arrow and yellow arrows). (*D*) Virtual reality (VR) rendering depicts the aneurysm with improved spatial and anatomical appreciation, the RCA (red) is positioned along the right atrioventricular groove. The right atrium (RA) and right ventricle (RV) are shown in transparent blue. (*E*) VR rendering of a 16-year-old girl with Kawasaki disease showing a large left anterior descending (LAD) coronary artery aneurysm (yellow arrows). The aorta and coronary arteries are shown in red; the RV in transparent blue; and the left ventricle (LV) in light purple. (*F*) VR rendering of a 14-year-old boy with Kawasaki disease demonstrates a thrombosed LAD aneurysm (purple; blue arrow), and a left internal mammary artery (LIMA) graft (yellow). The graft anastomosis with the distal LAD is indicated by the white arrow. The aorta, aortic branches and coronaries are shown in red, the pulmonary arteries in light blue; superior vena cava (SVC) in light purple.

**Figure 2:**
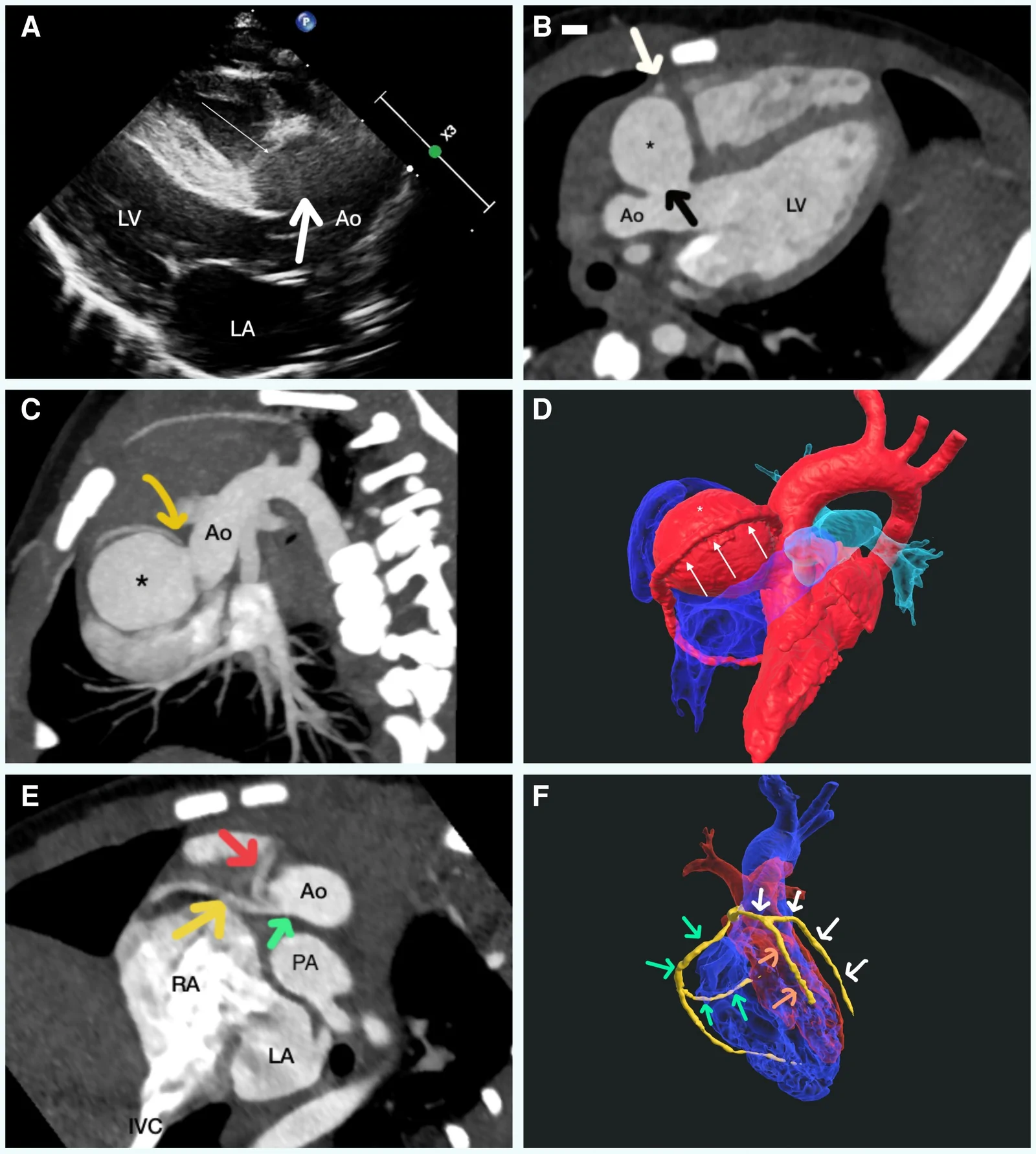
Newborn coronaries in context. This figure illustrates the application of VR in the assessment of newborn coronary anatomy. A newborn infant with a right aortic sinus of Valsalva (SoV) aneurysm diagnosed on antenatal and postnatal echocardiography is shown in panels *A–D*. (*A*) Postnatal echocardiography demonstrates an aneurysm arising from the sinus of Valsalva (thin white arrow) with a wide neck (thick white arrow). (*B*, *C*) Postnatal CT confirms a large sinus of Valsalva aneurysm (asterisk*) with a wide neck (black arrow) opening just above the right coronary cusp of the aortic valve. The right coronary artery (white arrow) arises from the aneurysm (*) with mild stenosis at its origin (yellow arrow) which is much better demonstrated on CT than ECHO. **Ao:** aorta. **LV:** left ventricle (*D*) Virtual reality rendering shows the proximal RCA (solid white arrows) wrapping around the aneurysm (*) which has protruded into the right atrioventricular groove, causing marked splaying of the adjacent right atrium and right ventricle. (*E*)A newborn with complete transposition of the great arteries. CT demonstrates a single-ostium coronary pattern, with the right coronary artery (yellow arrow) and the left anterior descending artery (red arrow) arising from a single ostium in the right facing sinus (green arrow). **Ao**: aorta **PA:** pulmonary artery **RA:** right atrium **LA:** left atrium (*F*) VR rendering again demonstrates the single ostium origin. RCA (green arrows) and LAD (white arrows). The transparency of the ventricles and great vessels demonstrate a right dominant coronary pattern. The LAD anterior to the aorta and giving rise to a branch supplying the right ventricle (orange arrows). Coronaries: yellow; Aorta and RV: blue, pulmonary arteries and LV: red.

**Figure 3:**
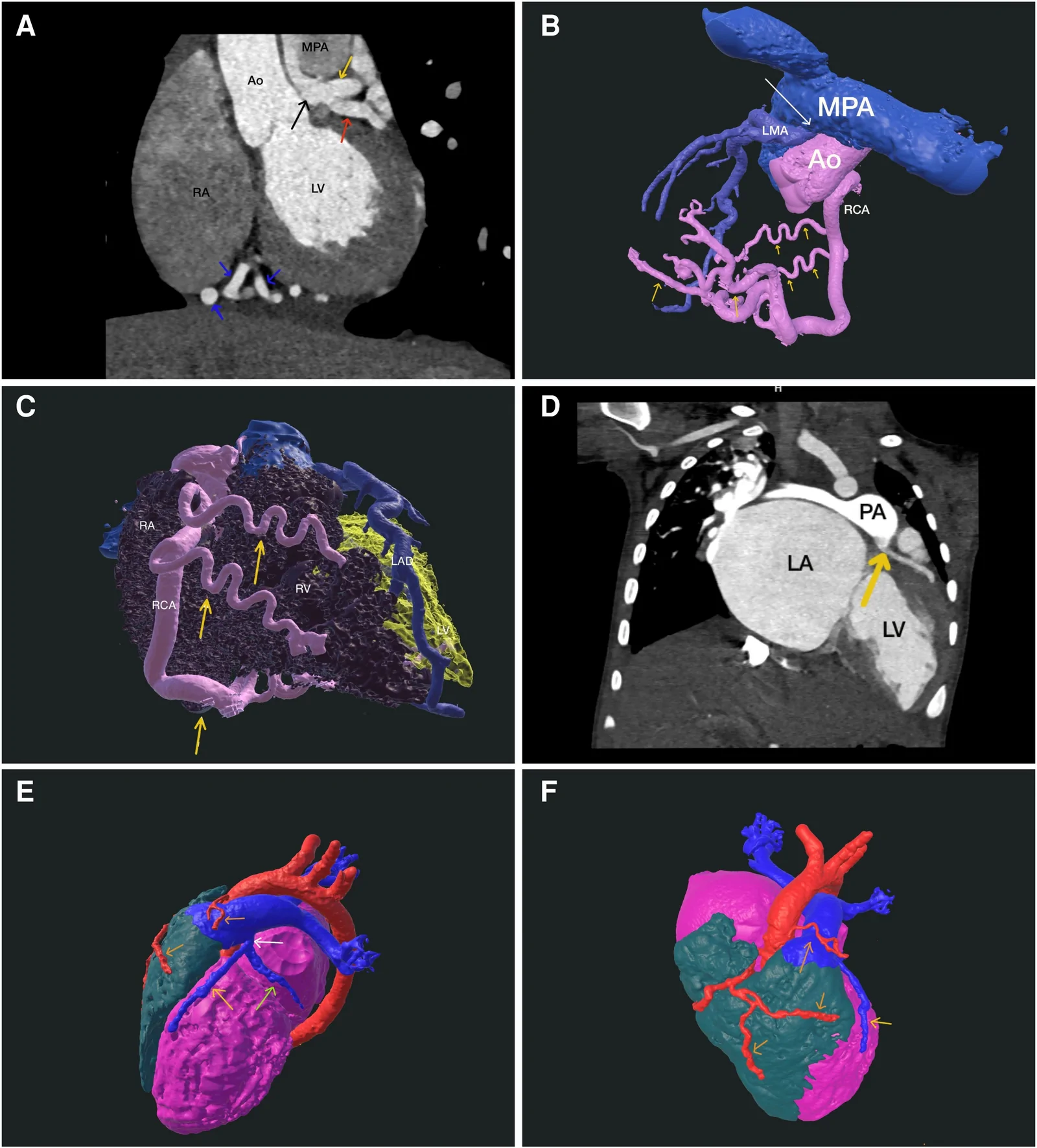
ALCAPA in 3D: from pulmonary origin to epicardial collateral network. A 13-year-old girl who presented with palpitations. (*A*) Computed tomography demonstrated anomalous left coronary artery from the pulmonary artery, with the left main coronary artery (LMA; black arrow), left anterior descending artery (LAD; yellow arrow), left circumflex artery (LCx; red arrow), and collateral vessels (blue arrows) identified. **Ao**: aorta **MPA:** main pulmonary artery **RA:** right atrium **LV:** left ventricle (*B*) A posterior view of the virtual reality rendering demonstrates the dilated left coronary arteries arising from the MPA (white arrow) and multiple collaterals (yellow arrows) from the right coronary artery (yellow arrows). Aorta, RCA and its branches: pink; Pulmonary arteries, LMA and its branches: blue (*C*) A second virtual reality view demonstrates the extensive epicardial collateral network (yellow arrows) RV: transparent purple; LV: transparent yellow. (*D*) An 11 month old infant who presented with failure to thrive. CT showed the origin of the LMA (yellow arrow) from the main pulmonary artery (PA) and a markedly dilated left atrium (LA) (*E*,*F*) Virtual reality images provide enhanced depth perception and spatial definition, demonstrating the LMA(white arrow), LAD(yellow arrow) and LCx (green arrow) as well as collateral vessels from the RCA(orange arrows). Pulmonary arteries, LMA, LCx, LAD: blue; aorta, RCA and branches: red; right ventricle: green, left ventricle and left atrium: pink.

All 7 cases could be successfully processed for immersive review (*Table 1*). Virtual reality was most helpful when the key questions were related to morphological alteration and spatial orientation of the coronary arteries. These are well illustrated in the delineation of coronary artery aneurysms in children with a history of Kawasaki disease (*Figure 1*), neonates with coronary artery abnormalities (*Figure 2*), and ALCAPA (*Figure 3*). Segmentation averaged approximately 2 h in infants vs. 1 h in teenagers as the smaller vessels in infants required more manual editing.

**Table 1:** Case characteristics, CT acquisition and virtual-reality assessment

Sex	Age	Diagnosis	History	CT acquisition/anaesthesia	Scan HR (bpm)	Coronary depiction	Structures depicted	VR segmentation time (min)	Perceived added value of VR
M	8 mo	Kawasaki	presented with prolonged fever and diagnosed Kawaki's disease clinically. Coronary artery aneurysm development despite treatment with IVIG and NSAIDs. Echo failed to see the whole RCA aneurysm hence CT performed.	retrospective ECG gated; GA with breath suspension	86	Aneurysm morphology, longitudinal extent and right AV-groove course	Aortic root, RCA, RA, RV	95	Global depiction of the aneurysm’s longitudinal extent and course in the right atrioventricular groove relative to the right-sided chambers.
M	2 d	Sinus of Valsalva aneurysm	Antenatally diagnosed Sinus of Valsalva aneurysm. CT for further delination postnatal.	retrospective ECG gated; GA with breath suspension	122	RCA origin and proximal course around the aneurysm	Aortic root, SoV aneurysm, proximal RCA, RA, RV	130	Clear depiction of the RCA arising from and wrapping around the aneurysm, with associated distortion of the adjacent right-sided chambers.
F	2 d	TGA with single coronary origin, with RCA and LMCA from right posterior facing sinus	Antenatally diagnosed TGA, post natal ECHO unable to visualised coronary origins clearly, CT performed	retrospective ECG gated; GA with breath suspension	127	Single origin, branching pattern and proximal coronary courses	Great arteries, ventricles, coronary ostium, RCA, LAD	140	Clarification of the single-ostium branching pattern and coronary courses relative to the transposed great arteries, relevant to coronary transfer.
F	11 mo	ALCAPA	Presented with severe mitral regurgitation, dilated LA, LV. Echo suspected ALCAPA confirmed by CT.	retrospective ECG gated; GA with breath suspension	98	Pulmonary origin of left main coronary artery, proximal branching and major RCA collaterals	MPA, aorta, LMCA, LAD, LCx, RCA, chambers	100	Improved spatial definition of the left main pulmonary origin, LAD–LCx branching and RCA collateral vessels within compact infant anatomy.
F	13 y	ALCAPA	Presented with recurrent palpitation. Echo found epicardial vascular structures and suspected coronary artery fistula. CT diagnosed ALCAPA	prospective ECG gated; no anaesthesia	60	Pulmonary origin and extensive epicardial collateral network	MPA, aorta, LMCA, LAD, LCx, RCA, ventricles	70	Integrated display of the anomalous pulmonary origin, dilated left coronary system and extensive epicardial RCA collateral network.
F	16 y	Kawasaki	presented as atypical kawasaki with myocarditis, limbic encephlitis and dilated coronary arteries	high pitch helical ECG gated; no anaesthesia	57	LAD aneurysm location and morphology	Aorta, left coronary system, LAD, ventricles	50	Intuitive localisation of the aneurysm within the coronary tree and along the anterior interventricular course of the LAD
M	14 y	Kawasaki with LIMA graft	presented with exertional chest pain and Echo showed giant coronary aneurysms with thrombosis in LAD	Prospective ECG gated; no anaesthesia	62	Native aneurysm, graft course and distal anastomosis	Native coronaries, thrombosed LAD aneurysm, LIMA graft, distal LAD	80	Unified visualisation of the thrombosed native aneurysm, LIMA graft course and distal graft anastomosis.

ALCAPA, anomalous left coronary artery from the pulmonary artery; AV, atrioventricular; bpm, beats per minute; CT, computed tomography; d, days; ECG, electrocardiography; ECHO, echocardiography; F, female; GA, general anaesthesia; HR, heart rate; IVIG, intravenous immunoglobulin; LA, left atrium; LAD, left anterior descending coronary artery; LCA, left coronary artery; LCx, left circumflex coronary artery; LIMA, left internal mammary artery; LMCA, left main coronary artery; LV, left ventricle; M, male; min, minutes; mo, months; MPA, main pulmonary artery; NSAIDs, non-steroidal anti-inflammatory drugs; RA, right atrium; RCA, right coronary artery; RV, right ventricle; SoV, sinus of Valsalva; TGA, transposition of the great arteries; VR, virtual reality; y, years.

## Discussion

This case series suggests that VR rendering is feasible for structures as small as paediatric coronary arteries. The high feasibility in this selected cohort was related to inclusion only of high-quality CT datasets with adequate vascular opacification, acquisition using tailored paediatric protocols,^6,7^ and selection by a trained imager of the motion-free cardiac phase that best demonstrated the lesion. Lesion conspicuity and depiction may vary across the cardiac cycle and depend on both the phase analysed and the timing of scan acquisition relative to the cardiac cycle. As only CT examinations of sufficient quality for coronary segmentation were selected, this study was not designed to compare image quality between acquisition techniques, and the findings may not reflect technically suboptimal examinations encountered in routine practice.

The small calibre of infant coronary arteries required longer segmentation time. Segmentation of coronary arteries of infants and small distal branches was technically demanding and often required manual editing, hence occasionally producing mildly irregular vessel contours. Minor surface irregularity was accepted when it reflected the limits of source-image resolution and did not affect the key anatomical relationships being demonstrated. In our series, the most consistent strengths were demonstration of coronary origins, proximal course, aneurysmal morphology, and larger collateral pathways. These represent scenarios in which clinicians must determine not only the presence of an abnormality, but also its spatial relationship to the aortic root, pulmonary artery, and atrioventricular grooves^1–3,8–10^ In paediatric coronary artery assessment, VR is likely to be most appropriate when CT image quality is high and when the anticipated benefit is improved spatial communication for multidisciplinary review, education, or procedural planning.^2–5^ Conversely, VR should not be regarded as a means of compensating for inadequate source imaging; rather, it enhances the interpretive value of well-acquired CT datasets. This distinction is particularly important when complex coronary findings are reviewed in a multidisciplinary setting where pre-procedural discussions are made by attending radiologists, cardiologists, and surgeons.

Practical implementation is primarily constrained by the time burden of segmentation, particularly for small coronary vessels. Segmentation is operator-dependent and becomes faster with experience, reflecting a learning curve. In our practice, integration into routine workflow became feasible only after recruitment of a dedicated radiographer. Software, hardware and training costs remain additional barriers to wider adoption. Further studies to determine whether incorporation of VR platforms in reviewing coronary artery anatomy would change decisions, reduce uncertainty, or improve workflow are warranted.

## Conclusion

In this small series, the principal practical contribution of VR was the conversion of high-quality CT data into an interactive three-dimensional representation of coronary anatomy. This appeared most valuable in cases where the coronary origin, proximal course, and spatial relationships of the abnormality were adequately captured by CT but were difficult to appreciate intuitively on conventional imaging views.

## Data Availability

No new data were generated or analysed in support of this research.

## Lead author biography

Dr. Carol Wing Kei Ng is a paediatric radiologist and clinical lead in cardiovascular imaging at Hong Kong Children’s Hospital. She is a European Association of Cardiovascular Imaging (EACVI) certified Level 3 congenital cardiac magnetic resonance imager and holds the European Diploma in Paediatric Radiology. With training in both paediatrics and radiology, including cardiac imaging fellowship training at Great Ormond Street Hospital, her interests include congenital cardiac CT and MRI, 4D flow imaging, 3D printing, virtual reality, and advanced imaging for surgical and interventional planning.
